# Factors Influencing Buprenorphine Prescribing among Physicians in New York State

**DOI:** 10.1155/2019/7832752

**Published:** 2019-12-18

**Authors:** Leslie A. Marino, Aimee N. Campbell, Edward V. Nunes, Lloyd I. Sederer, Lisa B. Dixon

**Affiliations:** ^1^Division of Behavioral Health Services and Policy Research, Columbia University, Department of Psychiatry, New York, NY, USA; ^2^Division of Substance Use Disorder, Columbia University, Department of Psychiatry, New York, NY, USA; ^3^Columbia/Mailman School of Public Health, New York, NY, USA

## Abstract

**Background:**

Increasing access to buprenorphine is an important strategy for curtailing the opioid epidemic. Research is needed to understand what facilitates prescribing among waivered physicians and how to increase the willingness and capacity to prescribe. This study describes prescribing patterns in a sample of buprenorphine-waivered physicians in New York (NY) in 2016 and examines factors influencing prescribing capacity among waivered providers.

**Methods:**

Surveys were mailed to a random sample of 300 physicians with DEA waivers to prescribe buprenorphine in NY which assessed demographics, practice characteristics, buprenorphine prescribing patterns, and barriers/facilitators to prescribing buprenorphine. Analyses include simple logistic regression to calculate the odds ratio, 95% confidence intervals, and *p* values, respectively, to examine differences in individual predictors among physicians that were actively prescribing buprenorphine and those that were not.

**Results:**

91 physicians responded to the survey, and 65% indicated they were currently prescribing buprenorphine. The mean patient census among physicians waivered to prescribe to 30 patients was 9.6 (SD = 9.7, median = 5), and to 100 patients, it was 60.5 (SD = 38.9, median = 72.5). Common facilitators included access to psychosocial referrals and better reimbursement, while inadequate resources, lack of time, and prior authorizations were the most common barriers.

**Conclusions:**

In addition to increasing the number of waivered physicians, policy-makers should provide enhanced training and implementation support for waivered physicians to start prescribing and facilitate continued and expanded prescribing among those already doing so.

## 1. Introduction

Medications for Opioid Use Disorder (MOUD), including methadone and buprenorphine, are among the most effective treatments. Both medications were placed on the World Health Organization's model list of “essential medicines” in 2009 [1], and the extensive use of MOUD in combination with a coordinated public health response was associated with a decrease in heroin overdose deaths in the 2000s [2]. Despite evidence of its effectiveness, few individuals with OUD can access medication, and the treatment-needed versus treatment-received gap for MOUD is estimated to be at least 1.4–1.5 million people [3].

Increasing access to buprenorphine is an important strategy for curtailing the opioid epidemic, but barriers exist among community-based physicians. Challenges to prescribing buprenorphine cited by community physicians in previous research across the U.S. include lack of staff time and training, perceived complexity of patients with OUD, poor reimbursement for office visits, and difficulty finding addiction specialists for consultation [4–7].

In response to the current public health crisis, substantial increases in federal and state funding have been ear-marked for enhancing access to medications for the treatment of OUD. Additional training and increased numbers of waivered prescribers are needed. However, training alone is not sufficient [8] unless the barriers that prevent physicians from taking on the treatment of OUD are better understood. Research is needed to understand what drives prescribing among waivered physicians and how best to increase and support the willingness and capacity to prescribe among those who do not.

This study aims to (1) describe the demographics and prescribing patterns of a sample of buprenorphine waivered physicians in New York State and (2) examine factors influencing prescribing of buprenorphine. Policy-makers and public health officials must better understand factors that can increase the likelihood that waivered physicians will prescribe buprenorphine to better respond to the current opioid epidemic.

## 2. Methods

### 2.1. Study Population

A list of buprenorphine waivered physicians in NYS as of March 2016 was purchased from the National Technical Information Service (http://www.NTIS.gov). The list included all physicians in NYS with a DEA number and provided the physician's name, the address where their DEA number is registered and whether they had an “*X*” number indicating they had received a waiver from the DEA to prescribe buprenorphine for 30 or 100 patients. There were 3,060 physicians with an active DEA number that also had an “*X*” number in NYS. From this list, a sample of 300 physicians was selected using a random number generator.

Physician recruitment took place between July and December, 2016. Physicians were recruited via mail at the address listed in the DEA database, and up to two additional attempts to contact the physician were made. They were sent a letter describing the purpose of the survey and explaining that participation in the survey amounted to consent to participate. The initial mailing included a copy of the survey to complete and return via mail with a prepaid envelope and a web address and QR code to scan with their phone or tablet which would bring them to an online version using the internet-based survey software, Qualtrics®. They received a $50 gift card for survey completion. Follow-ups were conducted via mail, phone call, and e-mail where available.

### 2.2. Measures

The survey questions were developed based on a review of the literature and expert consensus. The survey questionnaire included 22 questions divided into four sections: physician demographics, practice characteristics, buprenorphine prescribing patterns, and barriers/facilitators to prescribing buprenorphine. The barriers and facilitators were provided in a list with separate categories for “barriers” and “facilitators,” and respondents were instructed to “check all that apply.” Current prescribers were defined as those who stated they were currently prescribing buprenorphine to at least one patient. Nonprescribers were defined as those who were not currently prescribing, though they may have prescribed in the past. The mailing zip code was used to determine the county in which the physician was practicing.

### 2.3. Statistical Analysis

All analyses were conducted in 2017 with IBM SPSS Statistics for Windows, Version 24, Armonk, NY: IBM Corp. 2016. Descriptive statistics (mean, standard deviation or *n*, and proportion) were calculated for all survey variables and stratified by prescribing status. Physician age, number of years in practice, and number of other providers the physician knows that prescribe buprenorphine were treated as continuous variables. The remaining variables were categorical. Simple logistic regression was used to calculate the unadjusted odds ratio (OR), 95% confidence intervals (CI), and *p* value to explore differences in individual predictors among physicians that were actively prescribing buprenorphine and those that were not. All hypothesis tests were performed as 2-sided with level of significance 5%.

The study was approved by the study site Institutional Review Board.

## 3. Results

Ninety-one waivered physicians responded to the survey (30% response rate). The respondent sample was mostly male (70.3%) and white (70.3%), and the majority were practicing in New York City (57.1%) (Table 1). The mean age of providers was 52.0 (SD = 12.8), and the mean number of years they were in practice was 19.1 (SD = 13.0) (Table 1). Sixty-seven physicians (73.6%) were waivered to prescribe buprenorphine to 30 patients, while the remaining 24 were able to prescribe to 100 patients. Sixty physicians (65.9%) indicated they were currently prescribing buprenorphine, and the mean patient census among physicians waivered to prescribe to 30 patients was 9.6 (SD = 9.7, median = 5) and to 100 patients, it was 60.5 (SD = 38.9, median = 72.5) (data not shown). Most physicians (60%) reported knowing up to 5 other buprenorphine waivered providers, and only 12 (13%) reported not knowing anyone who prescribes buprenorphine (data not shown).

Providers who identified as non-White were significantly less likely to be currently prescribing buprenorphine (*p* value = 0.029). In addition, those physicians who primarily practice in nonaddiction specialties or in group practice settings (as compared with solo practice) were less likely to be currently prescribing buprenorphine (*p* values = 0.046 and 0.018, respectively; Table 1). Providers who were in practice longer, were not affiliated with an academic institution, and knew more providers who were also prescribing buprenorphine were significantly more likely to indicate that they were currently prescribing buprenorphine (*p* values = 0.022, 0.027, and 0.020, respectively; Table 1). Age, gender, geographic location, primary practice specialty, and perceived effectiveness of buprenorphine were not significantly associated with prescribing. Only 23% of providers indicated that they used state agencies for sources of informational support, including the New York State Department of Health (NYS DOH), the Office of Alcoholism and Substance Abuse Services (OASAS), or the state medical society for their profession, respectively (data not shown).

**Figure 1:**
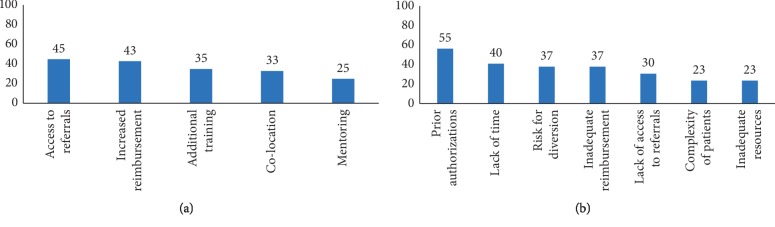
Factors influencing prescribing patterns among buprenorphine prescribers (*N* = 60). (a) Which of the following would enhance your ability to prescribe buprenorphine? (b) Which of the following barriers make it difficult for you to prescribe buprenorphine?

Access to psychosocial referrals for addiction and mental health treatment was an important facilitator of buprenorphine prescribing (45%), followed by increased reimbursement (43%) (Figure 1). Prior authorizations (55%) and lack of time (40%) were the most commonly listed barriers to prescribing buprenorphine. Many providers were concerned about the risk of diversion (37%), and the complexity of patients with opioid use disorders was also a barrier (23%). Inadequate resources, including both clinical resources (e.g., ability to perform urine toxicology in the office) and administrative resources/support, were also a common barrier (23%). Additional training (35%) was viewed as a potential facilitator of prescribing buprenorphine.

## 4. Discussion

At the time of this study, over 3,000 physicians in NYS had waivers to prescribe buprenorphine. In this sample, 65% of waivered physicians indicated they were currently prescribing buprenorphine and those who were waivered to prescribe to 30 patients were prescribing at low capacity, consistent with findings from other studies in the US [8, 9]. Many states are emphasizing the need to waiver more physicians, and federal legislation increased the patient limit to 275 patients per provider with approval from the DEA and SAMHSA after practicing at the lower thresholds for at least 1 year, but this study supports evidence that being waivered does not necessarily translate to active prescribing and suggests that more can be done to support providers who are already waivered [8, 10].

The race/ethnicity findings are unique. Few studies of physician perceptions and prescribing of buprenorphine have examined race/ethnicity as a factor so there is little data to which it can be compared. More than half of the non-white physicians in this study identified as Asian. One small study found that African-American physicians, regardless of their experience with prescribing buprenorphine, had more concerns around induction compared with white physicians, but this was attributed to artifact given the low number of African-American physicians responding to the survey [11]. It is possible that race/ethnicity may be a proxy for cultural or other related factors that have yet to be identified, and future research should include this factor to further explore this association.

The association between the number of physicians prescribing buprenorphine known to the respondent and increased likelihood of prescribing expands on previous research that demonstrated having a waivered partner increased the likelihood of prescribing [12]. This has important implications for physician training and capacity building. In addition, few prescribers indicated that they looked to state agencies for information and support. State agencies and state/regional/local chapters of medical societies can take a more proactive role in engaging physicians around prescribing buprenorphine and MOUD more broadly, not just by offering clinical guidelines but also giving physicians the opportunity to build collaborative networks with each other to provide support and expert consultation. Multilevel educational interventions which include academic detailing and clinical mentoring, along with building and strengthening physician networks for collaboration, have been shown to be effective strategies to increase physician likelihood of prescribing and their capacity to prescribe to more patients [13].

The factors influencing buprenorphine prescribing practices, including facilitators and barriers, were consistent with other studies [4, 7, 11, 12, 14, 15]. While some of these factors require policy changes at the state and federal levels (e.g., prior authorizations and improved reimbursement rates), many other factors (e.g., access to referrals sources, concerns about time, risk of diversion, patient complexity, and inadequate resources) can be addressed through ongoing training and implementation support after the providers complete the waiver training. This is consistent with what we know about medical education and continuing medical education. Physicians exposed to didactic training may gain knowledge, but they do not easily change their prescribing or practice behavior. Educational efforts that include practice and feedback (supervision, academic detailing, etc.) are more likely to get physicians to adopt a new practice [16, 17]. Further, facilitation on how to implement new practices in medical settings, taking into account integrated care models and systems-level change, may also support providers following training and education [18].

This study has several limitations. The study was restricted to physicians who practice in New York State, and given the large proportion of respondents from New York City (NYC), the generalizability to other states is uncertain. It is reassuring that many of the barriers and facilitators cited by respondents are similar to previous surveys of physicians. Thus, while some findings may depend on policies that vary state to state (i.e., insurance reimbursement and prior authorizations), other findings, such as those regarding training and support for physicians, are likely more generalizable to the workforce of physicians and other prescribers nationally. Second, there was a 70% nonresponse rate and those that did respond likely represent physicians who are current prescribers which may bias some of the findings. The sample was predominantly older, male, and White and may not generalize to the actual population of practicing physicians today, though it may be consistent with some studies that suggest waivered physicians are more likely to be older and male [19]. The small sample size of respondents limits some of the conclusions that can be drawn from the study.

## Figures and Tables

**Table 1:** Characteristics of buprenorphine prescribers versus nonprescribers.

Characteristics	Total *n* (%) or mean (SD) *n* = 91	Current prescribers *n* (%) or mean (SD) *n* = 60	Nonprescribers *n* (%) or mean (SD) *n* = 31	OR (95% CI)	*p* value
Age	52 (12.8) *N* = 83	53.9 (13.3) *N* = 55	48.1 (11.1) *N* = 28	1.04 (0.99–1.08)	0.055
Gender					
Male	64 (70.3)	42 (70.0)	22 (71.0)	Reference	
Female	27 (29.7)	18 (30.0)	9 (29.0)	1.05 (0.40–2.71)	0.924
Race/ethnicity					
White	66 (70.3)	48 (80.0)	18 (58.1)	Reference	
Non-white	25 (27.5)	12 (20.0)	13 (41.9)	0.35 (0.13–0.90)	0.029
Geographic location					
New York City	52 (57.1)	33 (55.0)	19 (61.3)	Reference	
Outside New York City	39 (42.9)	27 (44.5)	12 (38.7)	1.30 (0.54–3.14)	0.566
Years in practice	19 (13) *N* = 85	21.6 (13.7) *N* = 55	14.5 (10.3) *N* = 30	1.05 (1.01–1.10)	0.022
Primary practice specialty					
Psychiatry	44 (48.4)	25 (41.7)	18 (58.1)	Reference	
Medicine	35 (38.5)	28 (46.7)	8 (25.8)	2.52 (0.93–6.80)	0.068
Other	12 (13.2)	7 (11.7)	5 (16.1)	1.01 (0.28–3.69)	0.990
Addiction specialty					
Addiction specialist	17 (18.7)	15 (25.0)	2 (6.5)	Reference	
Nonaddiction specialist	74 (81.3)	45 (75.0)	29 (93.5)	0.21 (0.04–0.97)	0.046
Primary practice type					
Solo practice	30 (33.0)	25 (41.7)	5 (16.1)	Reference	
Group practice	61 (67.0)	35 (58.3)	26 (83.9)	0.27 (0.09–0.80)	0.018
Academic affiliation					
Yes	51 (56.7)	29 (48.3)	22 (73.3)	Reference	
No	39 (43.3) *N* = 90	31 (51.7) *N* = 60	8 (26.7) *N* = 30	2.94 (1.13–7.64)	0.027
Buprenorphine effectiveness					
Heavy/moderate	82 (90.1)	55 (93.2)	27 (93.1)	Reference	
Limited/none/DK	6 (6.6) *N* = 88	4 (6.8) *N* = 59	2 (6.9) *N* = 29	0.98 (0.17–5.70)	0.984
How many other buprenorphine providers do you know?	7.03 (8.5) *N* = 79	8.60 (10.0) *N* = 52	4.0 (2.8) *N* = 27	1.19 (1.03–1.38)	0.020

DK: do not know.

## Data Availability

Data used in this study will be available upon reasonable request to the corresponding author.
